# Modulation of spin-dependent diffraction based on dielectric metasurfaces

**DOI:** 10.1038/s41598-020-64943-z

**Published:** 2020-05-15

**Authors:** Yuanyuan Liu, Huiying Zhou, Jin Zhang

**Affiliations:** 1 College of Computer and Information Sciences, Central South University of Forestry and Technology, Changsha, 410004 China; 2 School of Electronic and Communication Engineering, Guiyang University, Guiyang, 550005 China; 3 School of Physics and Electronics, Hunan University, Changsha, 410082 China

**Keywords:** Optics and photonics, Optical physics

## Abstract

We propose theoretically and realize experimentally a tunable single-slit diffraction based on dielectric metasurfaces. Our dielectric metasurfaces can be regarded as polarization converters to generate inhomogeneous polarized light periodically variant in *x* direction. Different from the well-known single-slit diffraction of the scalar light field, our diffraction patterns exhibit two columns of diffraction fringes, which conceals spin-dependent splitting phenomenon. The underlying mechanism is attributed to the inherent nature of the Pancharatnam-Berry phase in the inhomogeneous polarized light. Interestingly, the spin-dependent splitting can be enhanced by increasing the polarization rotation rate of the inhomogeneous polarized beam or the transmission distance. Further, tunable diffraction phenomenon is observed with different slit widths or variant rotation angles of the dielectric metasurface and the slit. Our results may offer potential applications in spin-controlled nanophotonics.

## Introduction

Polarization, as an additional degree of freedom has been used to control and manipulate light fields^1^, which reveals nature of vector beams (inhomogeneous polarized light). Recently, the investigation of the new dimension has yielded different fields of research, such as generation of arbitrary vector beams^2–4^, analytical vectorial structure and propagation model^5,6^, intrinsic photonic spin Hall effect^7^, sub-wavelength localization^8^, nonlinear optics^9,10^, and laser micro-processing^11–13^. Motivated by the abounding benefits, researches have controlled the states of polarization by a metasurface^14–17^.

Diffraction plays a key role in demonstrating the wave nature of light. Single slit experiment includes main characteristics of diffraction phenomena and has given rise to a multitude of derivative experiments unveiling the wave nature of light, electrons, neutrons^18–20^. Recently, the diffraction of light fields with spatial phase distribution has yielded unexpected effects^21–24^. However, the single-slit diffraction of the inhomogeneous polarized light periodically variant in *x* direction with non-cylindrical symmetry is seldom referred to.

In this work, we demonstrate theoretically and explore experimentally spin-dependent diffraction of inhomogeneous polarized light with intrinsic Pancharatnam-Berry (PB) phase based on dielectric metasurfaces. By using dielectric metasurfaces, a horizontally linearly polarized beam is transformed into our desired inhomogeneous polarized light. Our diffraction patterns reveal spin-dependent splitting phenomenon. With the increase of the polarization rotation rate of the inhomogeneous polarized light or during beam propagation, the splitting becomes much stronger. Moreover, diffraction phenomenon is more distinct with the decline of the slit width. Furthermore, the diffraction patterns are tunable by rotating dielectric metasurfaces and the slit.

## Theoretical Model

A vector beam with polarization orientation periodically variant in *x* direction, whose elelctric field can be described by a Jones vector, has the form^25^

1 $$(\begin{array}{c}\cos \,\alpha \\ \sin \,\alpha \end{array})=\frac{\sqrt{2}}{2}({e}^{-i\alpha }|\,+\,\rangle \,+\,{e}^{i\alpha }|\,-\,\rangle )\mathrm{}.$$

**Figure 1:**
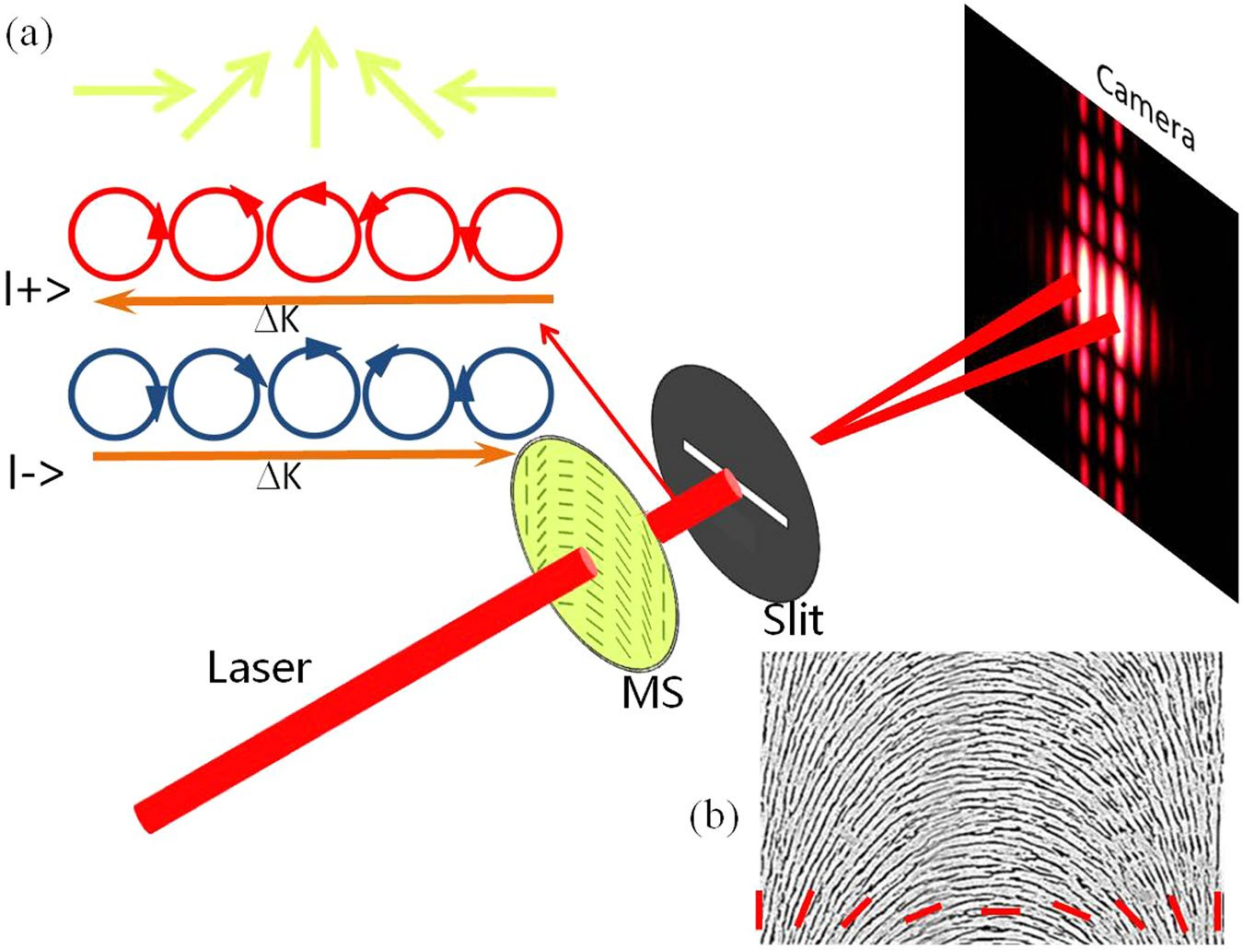
Schematic illustrating the single-slit diffraction of inhomogeneous polarization beam. The horizontally linearly polarized beam passing through the dielectric metasurface (MS) normally impinges into the single slit. The inhomogeneous polarization beam periodically variant in the *x* direction appears behind the dielectric metasurface. Then the spin-dependent diffraction patterns recorded by a camera occurs. Inset (**a**) Physical picture of the opposite PB phase gradient of the opposite spin states concealed in the inhomogeneous polarized light. Inset (**b**) SEM image of the metasurface over a period (*d* = 500 *μ*m) and its corresponding local optical axes (slow axis) orientation characterized by the red dashed lines.

Here, spin bases $|\,+\,\rangle$ and $|\,-\,\rangle$ represent left- and right-handed circular polarizations, respectively. Position-dependent function *α* = Ω*x* denotes inhomogeneous polarization, where Ω = *π*/*d* means the polarization rotation rate and *d* is the period of the vector beam. As an intrinsic property of vector beam, we could obtain the PB phase as $\Phi ={\sigma }_{\pm }\alpha$^7^, where ${\sigma }_{\pm }=\pm \,1$ are left- and right-handed circular polarizations, respectively. The relationship between the spin-dependent PB phase gradients of the opposite spin states is shown in Fig. 1(a). Consequently, the spin-dependent momentum shift is achieved based on the homogeneous gradient of the PB phase:

2 $$\Delta k=\frac{\partial \Phi }{\partial x}={\sigma }_{\pm }\Omega .$$

Importantly, the inhomogeneous polarization beam possesses a spin-dependent phase gradient as shown in Fig. 1(a), then induces real-space shift in *x* direction. The spin-dependent shift has the form^26,27^

3 $$\Delta x=\frac{\Delta k}{k}z=\frac{{\sigma }_{\pm }\Omega \lambda }{2\pi }z,$$

where *k* = 2*π*/*λ* indicates the wave number of the incident beam with *λ* the corresponding wavelength. We assume the initial position of the slit as the origin of the coordinate. *z* represents the distance from the single slit to the camera. It is easily observed that the splitting is enhanced upon beam propagation and increasing the polarization rotation rate Ω. In addition, spin-dependent splitting in position space could appear in the case of other underlying mechanisms^28–30^. It should be noted the spin-dependent splitting has exhibited important applications in precision metrology and imagine processing^31–33^.

Let us now consider how our desired inhomogeneous polarized light are generated by dielectric metasurfaces. In particular, the dielectric metasurface can be regarded as a polarization converter. A reliable fabrication method to obtain dielectric metasurfaces is via the femtosecond laser writing of spatially variant subwavelength nanogrooves in a fused silica glass. The femtosecond laser beam is focused 200 *μ*m below the surface of glass sample^14^. Based on intense laser irradiation, the silica glass sample (*SiO*_2_) decomposes into *SiO*_2(1−*x*)_ and *xO*_2_, whose refractive index is dependent upon laser intensity^34^. The metasurface is a uniaxial birefringent waveplate in nature. Importantly, it has a homogeneous phase retardation *δ* = 2*π*(*n*_*e*_ − *n*_0_)*h*/*λ*, where *h* is the writing depth, and *n*_*e*_, *n*_*o*_ represent the refractive indices of slow and fast waves, respectively. Their mathematic expressions are given as^35^

4 $${n}_{e}=\sqrt{\frac{{n}_{1}^{2}{n}_{2}^{2}}{f{n}_{2}^{2}+(1-f){n}_{1}^{2}}},\,{n}_{o}=\sqrt{f{n}_{1}^{2}+(1-f){n}_{2}^{2}}.$$

Here, *f* denotes the duty cycle, and *n*_1_ and *n*_2_ means the refractive indices of the two media that form the grating-like structure inside dielectric metasurface. Importantly, the difference between *n*_*e*_ and *n*_*o*_ represents the induced birefringence. In order to obtain our desired inhomogeneous polarized light, the dielectric metasurfaces with different rotation rates (Ω_*MS*_) *π*/500 rad*μ*m^−1^, *π*/750 rad*μ*m^−1^, *π*/1000 rad*μ*m^−1^ are fabricated. The dimension of grating-like structure area is engineered as 8 × 8 mm while dielectric metasurface has a diameter of 25.4 mm. For an operating wavelength of 632.8 nm with phase retardation *δ* = *π*, the writing depth is 70 *μ*m, the line width is 30–50 nm, and duty cycle is 0.1–0.2. Moreover, the dielectric metasurface is of a high transmission efficiency of 50.1% and a high conversion efficiency of 96.3% at 632.8 nm^36^. Its Jones matrix has the expression

5 $${\rm{T}}(x,y)=[\begin{array}{cc}\cos \,2\theta  & \sin \,2\theta \\ \sin \,2\theta  & -\,\cos \,2\theta \end{array}],$$

where *θ* is the orientation of the optical axis.

An input horizontally linearly polarized light with propagating in the *z* direction could be characterized by a Jones vector

6 $${{\bf{E}}}_{in}(x,y)={E}_{0}(x,y)(\begin{array}{c}1\\ 0\end{array}),$$

where ${E}_{0}(x,y)=\exp [-({x}^{2}+{y}^{2})/{w}_{0}^{2}]$ is a collimated Gaussian beam with *w*_0_ the beam waist. Next, the input beam impinges normally into the dielectric metasurfaces, we obtain the output beam ${{\bf{E}}}_{out}(x,y)=T(x,y){{\bf{E}}}_{in}(x,y)$ as

7 $${{\bf{E}}}_{out}(x,y)=\left(\frac{\cos \,2\theta }{\sin \,2\theta }\right){E}_{0}(x,y).$$

Compared with the inhomogeneous polarization shown in Eq. (1), we set *θ* = *α*/2. Therefore, the optical axis of the metasurface is also position-dependent and its rotation rate Ω_*MS*_ is the same as polarization rotation rate Ω.

Then the inhomogeneous polarized beam passes through a slit, the far field behind the single slit could be calculated by Fresnel diffraction formula^37^

8 $${{\bf{E}}}_{far}(x,y,z)=\frac{\exp (jkz)}{jz\lambda }\int {\int }_{-\infty }^{+\infty }\,{{\bf{E}}}_{out}(x,y)\cdot \exp \frac{jk}{2z}[{(x-{x}_{1})}^{2}+{(y-{y}_{1})}^{2}]d{x}_{1}d{y}_{1},$$

where *x*_1_(*y*_1_) axis is parallel to *x*(*y*) axis, and the *x*_1_*y*_1_ plane is set as the single slit plane. After substituting Eq. (7) into Eq. (8), the far field can be obtained as

9 $$\begin{array}{c}\begin{array}{rcl}{{\bf{E}}}_{far}(x,y,z) & = & \frac{\exp (jkz)}{jz\lambda }|\,+\,\rangle \int {\int }_{\,-\,\infty }^{\,+\,\infty }\,{E}_{0}(x,y)\exp (\,-\,i\alpha )\\  &  & \cdot \exp \frac{jk}{2z}[{(x-{x}_{1})}^{2}\,+\,{(y-{y}_{1})}^{2}]d{x}_{1}d{y}_{1}\\  &  & \,+\,\frac{\exp (jkz)}{jz\lambda }|\,-\,\rangle \int {\int }_{\,-\,\infty }^{\,+\,\infty }{E}_{0}(x,y)\exp (i\alpha )\\  &  & \cdot \exp \frac{jk}{2z}[{(x-{x}_{1})}^{2}\,+\,{(y-{y}_{1})}^{2}]d{x}_{1}d{y}_{1}.\end{array}\end{array}$$

Obviously, the polarization of the far field behind the single slit is same to that of the inhomogeneous polarized light. Based on the discussions above in Eqs. (1–3), the diffraction result can be viewed as superposition of two spin components, that is, spin-dependent splitting appears, which is attributed to the intrinsic PB phase *α* of inhomogeneous polarization light^7^. Therefore, our diffraction profile is spin-dependent as shown in Fig. 1. Especially, the slit is positioned close to the metasurface. Moreover, it is easily found that the splitting of the diffraction profile is much stronger with the increase of the propagation distance *z* or inhomogeneous rotation rate Ω.

## Results and Discussion

**Figure 2:**
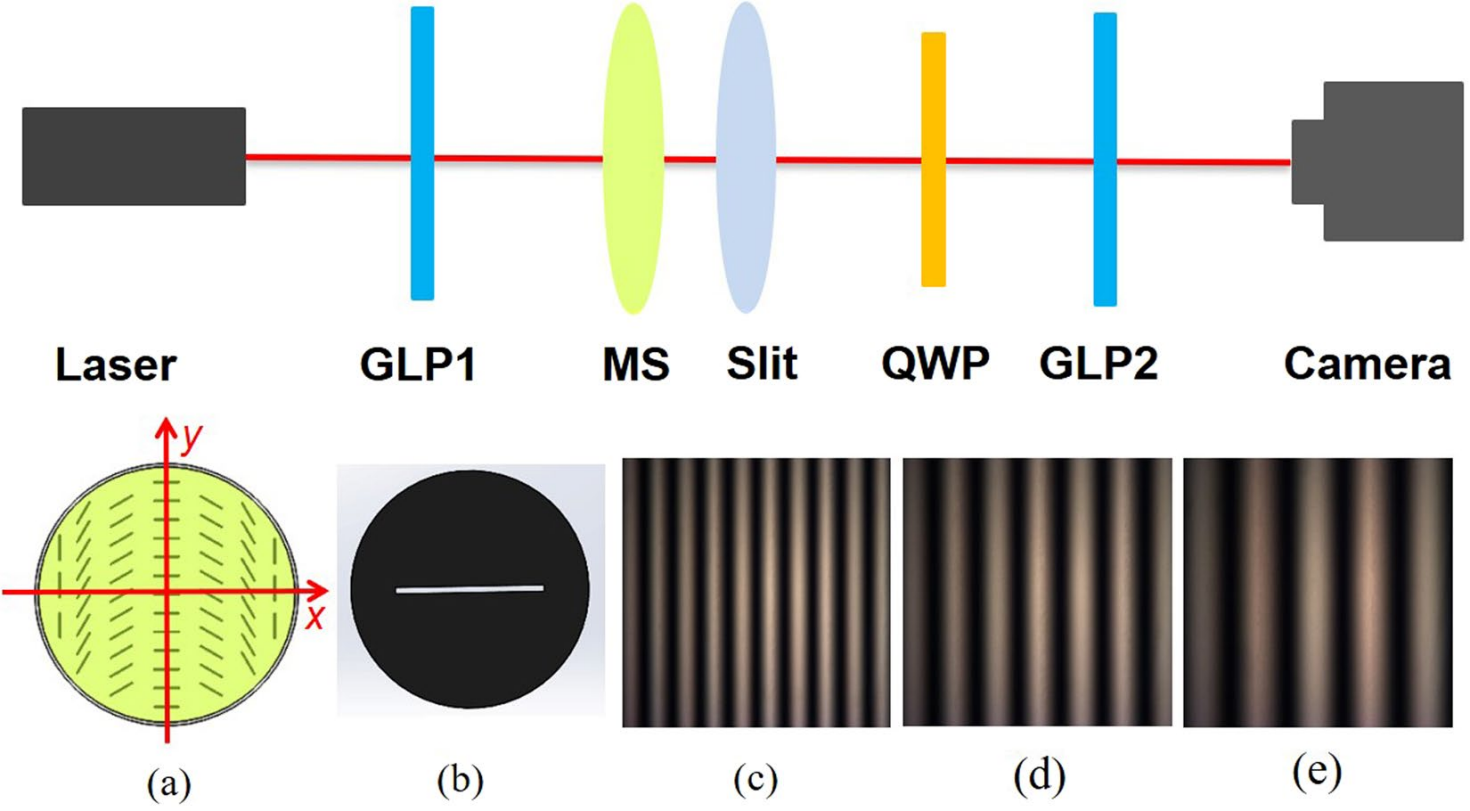
Experimental apparatus for observing the spin-dependent diffraction. Inset (**a**) Schematic picture of the dielectric metasurface with its fast axis orientation periodically variant in the *x* direction. Inset (**b**) Schematic picture of single slit. Inset (**c**–**e**) Cross-polarized images of optical axis spatial distribution in the dielectric metasurfaces applied in our experiments, with the corresponding rotation rates being *π*/500, *π*/750, *π*/1000 rad*μ*m^−1^, respectively, under cross linear polarizers.

We implement an experiment to realize the spin-dependent diffraction [Fig. 2]. A He-Ne laser with operation wavelength *λ* = 632.8 nm serves as the light source. The laser beam is transformed to a horizontally linear polarization state by the first Glan laser polarizer (GLP1). Then the horizontally linearly polarized beam normally impinges into the dielectric metasurface [Fig. 2(a)] and single slit [Fig. 2(b)], which is closely near the dielectric metasurface. There are three different single slits with widths 50 *μ*m, 100 *μ*m, 200 *μ*m, respectively. The cross-polarized images of the dielectric metasurfaces with different rotation rates (Ω_*MS*_) *π*/500 rad*μ*m^−1^, *π*/750 rad*μ*m^−1^, *π*/1000 rad*μ*m^−1^, are shown in Fig. 2(c–e) schematically, respectively. The polariscopic images reveal that the optical-axis spatial distributions are agreed with what Fig. 2(a) depicted and the phase retardance is *π* in the metasurface. In addition, the SEM image of the metasurface in Fig. 1(b) also means the local optical axes location is same to what Fig. 2(a) depicted. A quarter-wave plate (QWP) and another Glan laser polarizer (GLP2) cooperate with the camera to measure the Stokes parameter *S*_3_ distributions. In our experiment, the distance from the single slit to the camera *z* is assumed as 3 m. The *S*_3_ could characterize the circular polarization degree and be written as ${S}_{3}=({I}_{{\sigma }_{+}}-{I}_{{\sigma }_{-}})/({I}_{{\sigma }_{+}}+{I}_{{\sigma }_{-}})$^38^, where ${I}_{{\sigma }_{+}}$ and ${I}_{{\sigma }_{-}}$ are recorded output intensity of left- and right-handed circular polarization components, respectively. Via a series of data process, we calculate the *S*_3_ pixel by pixel.

**Figure 3:**
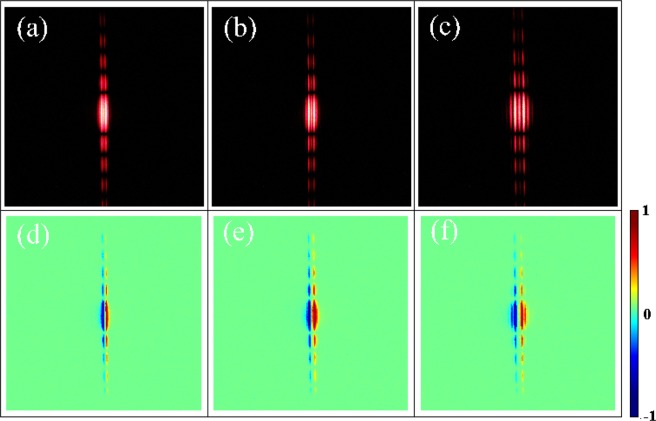
The intensity profile (upper panels) and corresponding *S*_3_ results (lower panels) after passing through the single slit. Here, we assume Ω = *π*/1000, *π*/750, *π*/500 rad*μ*m^−1^ and *w* = 100 *μ*m (from left to right). Red and blue indicate left- and right-circular polarized states, respectively.

Experimental diffraction patterns and corresponding *S*_3_ results of inhomogeneous polarization beams with Ω = *π*/1000 , *π*/750, and *π*/500 rad*μ*m^−1^ are presented in Fig. 3. Especially, the diffraction patterns are recorded by the camera without any data process. The diffraction profile above exhibit three columns of diffraction fringes. The middle column with a low intensity retains diffraction fringes of incident linearly polarized light, which is our well known single-slit diffraction of the scalar light field. This column means the unconverted portion of incident photons without any phase modulation. The possible reason is that our metasurface is not a perfect half-wave plate at the wavelength of *λ* = 632.8 nm. The left and right columns represent right- and left- circular polarizations, respectively, which is revealed by calculating the Stokes parameter *S*_3_. Therefore, our diffraction is referred to as spin-dependent diffraction, which perfectly verifies Eq. (9). Evidently, with the variant rate of inhomogeneous polarization beam Ω increasing, the spin-dependent splitting is enhanced, which agrees well with Eq. (3).

**Figure 4:**
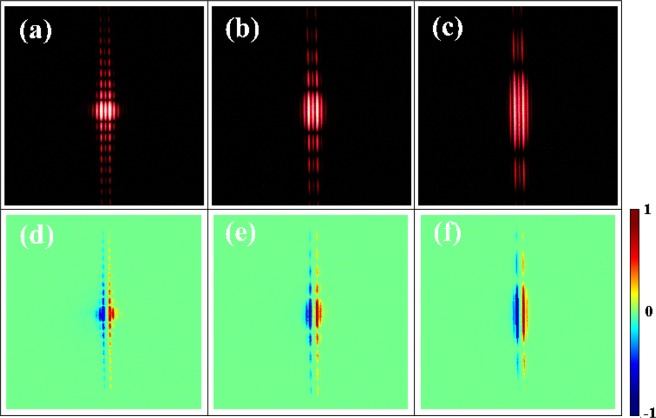
The intensity profile (upper panels) and corresponding *S*_3_ results (lower panels) after passing through the single slit. Here, we set *w* = 200, 100, 50 *μ*m and Ω = *π*/500 rad*μ*m^−1^ (from left to right). Red and blue indicate left- and right-circular spin states, respectively.

Then we consider the influence of slit width *w* on the spin-dependent diffraction. The experimental intensity profile and corresponding *S*_3_ results for *w* = 200, 100, 50 *μ*m are shown in the upper panels and lower panels of Fig. 4. The spin-dependent splitting is agreed well with Eq. (9). Furthermore, the diffraction phenomenon is more evident with the decline of slit width *w*. This is because when the order of magnitude of slit width is near the incident wavelengths, the slit width is smaller, or the incident wavelength is bigger, the diffraction phenomenon is more significant^38^.

**Figure 5:**
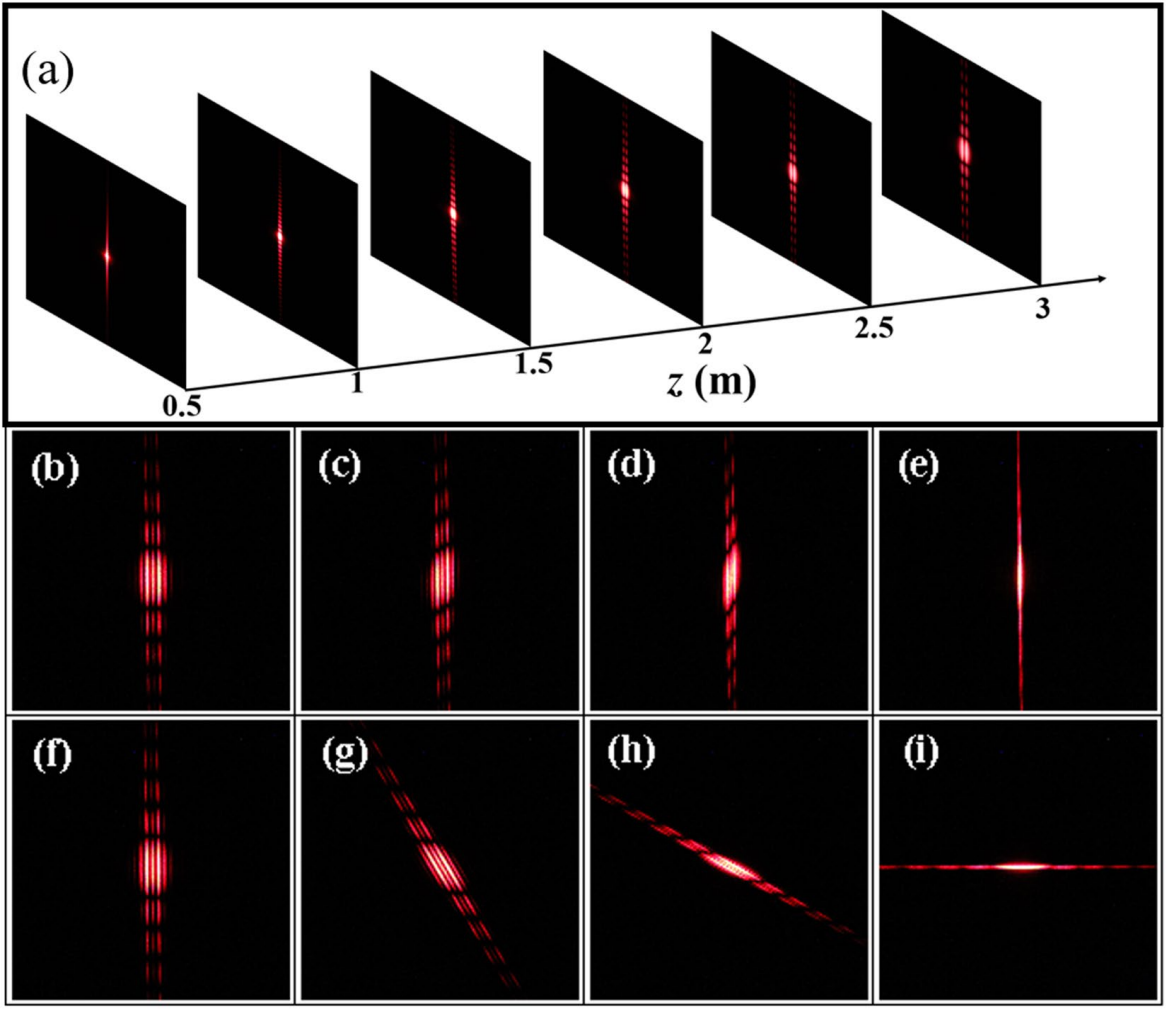
(**a**) The intensity patterns recorded by the camera with different transmission distances *z*. (**b**–**e**) The intensity profile observed in the case of the dielectric metasurface rotated through *β* = 0°, 30°, 60°, and 90°, respectively, and the slit placed in *x* direction. (**f**–**i**) The intensity profile observed in the case of the slit rotated through *γ* = 0°, 30°, 60°, and 90°, respectively, and the metasurface placed in *x* direction. We assume *w* = 100 *μ*m and Ω = *π*/500 rad*μ*m^−1^.

As aforementioned, the intrinsic PB phase gradient of our inhomogeneous polarization light results in an angular shift in momentum space, then the real-space shift increasing linearly with the transmission distance *z* appears, so the inhomogeneous polarization beam during propagation is instable. As a demo, we measure the diffraction patterns during beam propagation under *w* = 100 *μ*m and Ω = *π*/500 rad*μ*m^−1^. The experimental results are shown in Fig. 5(a). The diffraction profile gradually splits into two columns of diffraction fringes when transmission distance *z* increases, which perfectly verifies Eq. (9) and Eq. (3).

Furthermore, we explore the influence of rotating the dielectric metasurface and the slit on diffraction patterns, respectively. All discussions before are based on both the dielectric metasurface and the slit arranged in *x* direction, as exhibited in Fig. 5(b,f), respectively. The splitting phenomenon perfectly verifies the theoretical analysis in Eq. (9). Rotating the dielectric metasurface merely, processed results for various rotation angles *β* of the metasurface are obtained as depicted in Fig. 5(b–e). It is distinct that the centers of the diffraction patterns retain the same angle with the rotation angle *β* of the metasurface. The direction of the inhomogeneous polarization beam in our experiment is along rotation angle *β* of the metasurface, which gives a quantitative explanation for the rotation of centers as shown in Fig. 5(b–e). Especially, for *β* = 90° [Fig. 5(e)] the splitting phenomenon vanishes because the spin-dependent splitting in *y* direction induced by the inhomogeneous polarization beam is concealed. Under rotating the slit merely, processed results for different rotation angles *γ* of the slit are recorded in Fig. 5(f–i). Unambiguously, diffraction fringes retain being perpendicular to varying rotation angles *γ* of the slit. Note that there is no splitting phenomenon for *γ* = 90 [Fig. 5(i)], which is attributed to generated inhomogeneous polarization beam in *x* direction. Moreover, we can see that rotation angle *β* and *γ* play different roles in steering diffraction phenomenon.

## Conclusion

In conclusion, we have investigated, both theoretically and experimentally, the peculiar behaviors of the single-slit diffraction of inhomogeneous polarized beam based on dielectric metasurfaces. The diffraction patterns reveal the spin-dependent splitting phenomenon. We also find that this splitting originates from the intrinsic Pancharatnam-Berry phase gradients of the inhomogeneous polarization. Moreover, the increasing of the polarization rotation rate of inhomogeneous polarized beam or the transmission distance induces more significant splitting. Further, we can vary slit widths or rotation angles of inhomogeneous polarized beam and the slit to obtain tunable diffraction phenomenon. Our results are expected to hold potential applications in spin-controlled nanophotonics.

## Data Availability

No datasets were generated or analysed during the current study.
